# Novel Advances in Modifying BMPR2 Signaling in PAH

**DOI:** 10.3390/genes12010008

**Published:** 2020-12-23

**Authors:** Svenja Dannewitz Prosseda, Md Khadem Ali, Edda Spiekerkoetter

**Affiliations:** 1 Division Pulmonary, Allergy and Critical Care Medicine, Department of Medicine, Stanford University, Stanford, CA 94305, USA; svenja.dannewitz@gmail.com (S.D.P.); mdali@stanford.edu (M.K.A.); 2 Vera Moulton Wall Center for Pulmonary Vascular Diseases, Stanford, CA 94305, USA; 3 Institute for Experimental and Clinical Pharmacology and Toxicology, Albert-Ludwigs University Freiburg, 79104 Freiburg, Germany

**Keywords:** PAH, pulmonary hypertension, bone morphogenetic protein receptor 2, signaling, repurposed drugs, pharmaceuticals, miRNA, clinical trials

## Abstract

Pulmonary Arterial Hypertension (PAH) is a disease of the pulmonary arteries, that is characterized by progressive narrowing of the pulmonary arterial lumen and increased pulmonary vascular resistance, ultimately leading to right ventricular dysfunction, heart failure and premature death. Current treatments mainly target pulmonary vasodilation and leave the progressive vascular remodeling unchecked resulting in persistent high morbidity and mortality in PAH even with treatment. Therefore, novel therapeutic strategies are urgently needed. Loss of function mutations of the Bone Morphogenetic Protein Receptor 2 (BMPR2) are the most common genetic factor in hereditary forms of PAH, suggesting that the BMPR2 pathway is fundamentally important in the pathogenesis. Dysfunctional BMPR2 signaling recapitulates the cellular abnormalities in PAH as well as the pathobiology in experimental pulmonary hypertension (PH). Approaches to restore BMPR2 signaling by increasing the expression of BMPR2 or its downstream signaling targets are currently actively explored as novel ways to prevent and improve experimental PH as well as PAH in patients. Here, we summarize existing as well as novel potential treatment strategies for PAH that activate the BMPR2 receptor pharmaceutically or genetically, increase the receptor availability at the cell surface, or reconstitute downstream BMPR2 signaling.

## 1. Introduction

Pulmonary Arterial Hypertension (PAH) is a cardio-pulmonary-vascular condition, where a progressive occlusion of the distal pulmonary vasculature leads to an increase in pulmonary vascular resistance and right ventricular (RV) afterload, resulting in RV failure and premature death [1,2]. Histopathological analysis suggests that dysfunction of key cellular components of the pulmonary vasculature, namely endothelial and smooth muscle cells, pericytes, inflammatory cells, and adventitial fibroblasts, induce pulmonary vascular remodeling [3,4]. This results in narrowing of the vessel lumen and formation of complex vascular lesions, which together raise pulmonary vascular resistance, increasing pulmonary arterial pressure as well as the afterload for the right ventricle.

Although PAH is a rare disease affecting only about 1–2 of every 1 million individuals annually, the mortality and morbidity rate is high and, if untreated, PAH quickly leads to right ventricle failure and death after 2–3 years [5,6]. PAH may be heritable (with a family history of PAH), idiopathic (without a family history, unknown cause), or associated (linked to interstitial lung disease, congenital heart disease, autoimmune disease, etc.) [7]. Whilst the exact cause of PAH is not known, genetic factors (mutations or epigenetic changes), environmental factors (e.g., hypoxia, viral infections, anorectic agents, stimulants, etc.) and immune or inflammatory triggers may contribute to the cause or progression of the disease [4]. Importantly, there is no cure for PAH. Existing drugs target pulmonary vasodilation, proliferation and endothelial function by increasing nitric oxide (NO), inhibiting endothelin and voltage-gated calcium channels and by augmenting prostacyclin signaling pathways [8]. However, these drugs only partially increase survival and improve quality of life, while the majority of patients ultimately become resistant to medication and succumb to the disease [9]. With current treatments, the 5-year survival of PAH patients has been improved from 34% to 60%, yet these drugs are not capable of reducing the extent and progression of vascular and cardiac remodeling, resulting in eventual clinical deterioration of PAH patients over time [10]. 

Thus, new, effective and disease modifying therapies are urgently needed [11], therapies that target the underlying molecular mechanisms responsible for pulmonary vascular remodeling, which is the hallmark of PAH. Over the past two decades, many cellular and molecular mechanisms have been described as playing key roles in the pathogenesis of disease in preclinical and clinical settings [4,12]. Here, we focus on modulation of bone morphogenic protein receptor 2 (BMPR2) signaling [7] as a key mechanistic pathway and potential master switch in the pathogenesis of PAH.

## 2. The BMPR2 Signaling Pathway 

In 2000 two independent groups identified mutations in BMPR2 as causative for the familial form of PAH [13,14]. BMPR2 carriers with PAH have an earlier disease onset than idiopathic PAH patients [15]. Interestingly, male patients were more likely to possess a BMPR2 mutation than women and develop severe disease in presence of a BMPR2 mutation [16].

Meanwhile, researchers have identified mutations in over 16 genes in patient with hereditary PAH (HPAH) that may predispose to PAH, including BMPR2 of course, but also receptors that are part of or are interacting with the BMPR2 pathway such as activin A receptor type II-like 1 (ACVRL1), endoglin (ENG), caveolin-1 (CAV1), SMAD1, SMAD4, SMAD9, bone morphogenetic protein receptor type 1B (BMPR1B), eukaryotic translation initiation factor 2 α kinase 4 (EIF2AK4), and growth differentiation factor 2 (GDF2) [17]. While most identified gene mutations are relatively rare (1–3% cases), heterozygous loss-of-function mutations in the BMPR2 gene are the most common and occur in 53–86% of HPAH and 14%–35% of idiopathic PAH (IPAH) patients [18]. To date, more than 300 mutations, predominantly nonsense and frameshift types, have been identified in the BMPR2 gene in PAH patients. BMPR2, encoded by the *BMPR2* gene, is a member of the serine/threonine kinase transmembrane proteins belonging to the TGFβ receptor superfamily. BMPR2 binds BMP ligands such as BMP2, BMP4, BMP6, BMP7 and BMP9. BMPs typically play a role in a wide range of signal pathways involved in cellular differentiation, growth, and apoptosis and in embryogenesis, development, and tissue homeostasis. In the canonical BMP signaling pathway, upon binding of BMP ligands, BMP type 2 receptors (e.g., BMPR2 (ActRIIA) and ActRIIB)) recruit, complex and phosphorylate BMP type 1 receptors (e.g., Activin receptor-like kinase 1(ALK1), BMPR-1A (ALK3), BMPR-1B (ALK6), and ActR-1A (ALK2)), which then phosphorylate receptor-regulated SMADs (R-SMADs). These R-SMADs form a complex with co-SMADs (e.g., SMAD4) and translocate to the nucleus where the complex binds to a BMP response element DNA sequence. As a result, the complex acts as transcriptional regulator of target gene expression including Inhibitor of DNA Binding 1, 2, and 3 (ID1, ID2, ID3) or cyclin-dependent kinase inhibitor 1A and 2B (CDKN1A and CDKN2B) by binding to the BMP responsive element (BRE), which plays a critical role in cell proliferation, apoptosis and migration. In addition to the canonical SMAD mediated signaling pathway, several non-canonical BMP signaling pathways are also activated by BMPR2, including p38 Mitogen-Activated Protein Kinase (MAPK), Extracellular Signal-Regulated Kinase (ERK), Phosphoinositide 3-kinase (PI3K)/Akt signaling, Peroxisome proliferator-activated receptor γ (PPAR γ)/Apolipoprotein E (ApoE)/ High –density lipoprotein cholesterol (HDLC), Wingless (Wnt), Caveolin, Rho-GTPases, Protein Kinase C (PKC) signaling and NOTCH signaling [19].

## 3. Regulation of BMPR2 Signaling

A tight regulation of BMPR2 signaling is exerted by extracellular agonists and antagonists, such as the inhibitory molecule Noggin, Chordin and gremlin1 [20], which is upregulated by endothelin1 [21]. Intracellularly, a feedback loop controls BMPR2 signaling through the activity of inhibitory SMADs (iSMADs) SMAD6 and SMAD7, which inhibit the phosphorylation of SMAD2 and SMAD3, signaling molecules that function as counterparts to SMAD 1/5 signaling. SMAD1 degradation is initiated through SMURF1 and SMURF2 targeting, which downregulates further downstream gene expression [22]. BMPR2 downstream signaling is further regulated by FK binding protein 12 (FKBP12), which prevents the activation and phosphorylation of type 1 receptors in absence of a ligand [23] FKBP12 furthermore maintains the balance of rSMAD and iSMAD signaling, by regulating SMAD2/3 activity and recruiting SMAD7 [24].

The availability of BMPR2 receptors at the cell surface is provided by the balance of receptor expression and degradation, as well as receptor shuttling to the cell surface [25]. While upregulation of BMPR2 receptor expression has recently been explored as a therapeutic strategy in PAH [26], little is known about intracellular signaling molecules that target BMPR2 expression. We recently explored upstream modulators of BMPR2 expression and described two novel players in BMPR2 signaling that can increase BMPR2 expression, namely Fragile Histidine Triad (FHIT) and lymphocyte-specific protein tyrosine kinase (LCK) [27].

In contrast to the lack of data on the positive regulation of BMPR2 expression, the mechanisms of its downregulation are well-described, whereas regulation via micro RNAs (miRs) and receptor degradation play major roles. miR-20a and miR17 have both been connected to the downregulation of BMPR2 expression [28,29] whereas the miR17-92 cluster downregulated BMPR2 by engaging the inflammatory cytokine IL-6 via STAT3 [30]. Hypoxia downregulates BMPR2 signaling through miR-21 and miR-125a [31]. miR-302 targets BMPR2 signaling in PASMCs, thereby reducing their proliferation [32] miR21 is connected to a feedback inhibition of BMPR2 signaling, as its expression is induced by BMPR2 signaling on the one hand, but also reduces BMPR2 expression in PAECs. Therefore, the lack of its expression in vivo induces PH, while the use of miR-21 inhibitors in a rodent model of PH supports vascular regeneration in the hypoxia-remodeled pulmonary vasculature [33,34]. In addition to BMPR2 regulation by micro RNAs, it was recently described that 17-estradiol- induced binding of the estrogen receptor to the BMPR2 gene promotor, inhibited BMPR2 transcription, a finding that might explain the sex-based differences in PAH pathogenesis [35,36]. A reduction of BMPR2 receptor presence on the cell surface can be achieved by its premature degradation in connection to infection and inflammation. The inflammatory cytokine Tumor necrosis factor α (TNFalpha) activates metalloproteases that can cleave the receptor, and viral particles (i.e., Kaposi sarcoma-associated herpesvirus KSHV) can ubiquinate BMPR2, leading to its lysosomal degradation [37]. Furthermore, in the absence of BMPR2, SMAD signaling can shift from rSMAD-dominated signals of BMPR2 to the activation of the rSMADs SMAD2, SMAD3 and SMAD4, which are controlled by TGFβ [38] activating EC ITGB1 transcription, leading to EndMT, stress fiber production and actomyosin contractility.

Defective BMPR2 signaling caused by a mutational change in the BMPR2 gene can be rescued, as shown in unaffected BMPR2 mutant carriers through an effective feedback loop. When BMPR2 is functionally inactivated or reduced, the expression of receptor antagonists such as FKBP1A or Gremlin1 is reduced, while, similarly, cellular receptor activators are being upregulated [39].

## 4. BMPR2 Deficiency and Pulmonary Hypertension

Despite the high frequency of BMPR2 mutations in PAH patients, the disease penetrance rate is ~20% of the mutation carriers, suggesting that, in addition to BMPR2 mutations, other unidentified genetic, epigenetic, or environmental factors are involved in the development of the disease, potentially by decreasing BMPR2 expression and signaling activity below a specific threshold required to cause disease.

Furthermore, in PAH patients with and without BMPR2 mutations, BMPR2 expression and signaling activity is impaired in the pulmonary vasculature [40,41], suggesting that dysfunction of BMPR2 signaling is a key common feature in PAH patients. 

Pulmonary endothelial-specific deletion of BMPR2 in mice recapitulates human PAH features [42]. PAH manifestations are also observed in mice expressing a dominant-negative BMPR2 gene in pulmonary smooth muscle cells [43,44]. Similarly, haplo-insufficient BMPR2 mutant rats developed severe dysfunction of the cardio-pulmonary-vascular system, such as distal vessel muscularization, loss of microvascular vessels, inflammation, RV and endothelial dysfunction as well as intrinsic cardiomyocyte dysfunction [45].

Impaired BMPR2 signaling is associated with aberrant vascular cell phenotypes, including pulmonary arterial endothelial cells (PAEC) apoptosis, hyperproliferation and apoptosis resistance of pulmonary arterial smooth muscle cells (PASMC), and inflammation [3,12]. These findings suggest that targeting and thereby increasing BMPR2 expression and signaling could be an effective therapeutic approach for treating PAH.

## 5. Therapeutic Strategies to Modify BMPR2 Signaling

As outlined above, the mechanistic causes of BMPR2 deficiency in PAH can be defined as either receptor inactivation, decreased receptor expression, or an impairment of the receptor’s downstream signaling pathway [19]. In recent years, many novel approaches have emerged that target the BMPR2 pathway and are promising for clinical translation. Here, we have grouped and classified pharmacological and genetic interventions as follows: (a) targeting the BMPR2 receptor to increase its activity by pharmacological activators or gene-directed modulation, (b) increasing receptor availability at the cell surface by increasing signaling upstream of BMPR2, preventing receptor degradation or increasing the receptor shuttling to the cell surface, and (c) reconstituting BMPR2 downstream signaling by targeting interacting signaling pathways.

### 5.1. Targeting BMPR2 Receptor Activity

#### 5.1.1. Receptor Activation 

The activity of the BMPR2 receptor can be pharmaceutically increased by pharmacological activators, as long as a small quantity of BMPR2 exists and/or the potential of the BMPR2 protein to be activated is not prevented by the presence of a mutation in the BMPR2 gene. The most direct activation of BMPR2 signaling can be achieved pharmaceutically through the administration of recombinant BMP-9 ligand which has been proposed as a therapeutic strategy for use in PAH [26]. 

#### 5.1.2. Relieving Receptor Inhibition 

The inhibition of the BMPR2 receptor can be pharmacological or genetical in nature. The functional activity of the BMPR2 receptor complex can be repressed by the intracellular binding of FKBP12 to the intracellular domain of the type 1 transmembrane receptors activin receptor-like kinase 1 (ALK1), ALK2, and ALK3 and presence of the phosphatase Calcineurin, which binds to FKBP12. The release of FKBP12 from the receptor complex by BMPR2 ligands activates downstream (intracellular) BMPR2 signaling [23]. 

The activation of the receptor complex could therefore be induced by inhibitors of Calcineurin and compounds that bind FKBP12 themselves and prevent interaction with the type 1 receptors. Cyclosporine is an inhibitor of Calcineurin that decreased pulmonary arterial smooth muscle cell proliferation in vivo and apoptosis in vitro, while partially reversing the severity of experimental PH in monocrotaline treated rats [46]. Rapamycin, an FKBP12 ligand, has been shown to ameliorate the extend of artery smooth muscle cell proliferation [47]. The dual inhibition of both FKBP12 and Calcineurin was achieved by FK506(Tacrolimus) [23], which facilitates the release of the FKBP12/Calcineurin complex from the type 1 receptor by binding to both molecules, and thereby activating downstream canonical and non-canonical BMPR2 signaling even in presence of a BMPR2 mutation. FK506 was identified as the best BMPR2 activator in a high-throughput luciferase reporter assay of 3756 FDA-approved drugs using ID1 expression as the assay readout [23], superior to Rapamycin and Cyclosporine. In vitro, FK506 activated downstream BMPR2 signaling via SMAD1/5, MAPK and ID1 signaling in healthy PA endothelial cells (PAECS), while normalizing endothelial dysfunction in PAH PAECs. FK506 prevented experimental PH in BMPR2+/- mice and reversed PH in both the rat monocrotaline induced and Sugen-Hypoxia induced PH, whereby it reduced right ventricular systolic pressure (RVSP), right ventricular hypertrophy (RVH), pulmonary vascular medial hypertrophy and neointima formation. FK506 was found to be safe and well tolerated in a Phase 2a proof-of concept safety and tolerability study [48] and has shown promise as compassionate use in three end-stage PAH patients [49].

#### 5.1.3. Gene-Directed Modulation of the BMPR2 Receptor

The promise of genetic interventions to correct a specific BMPR2 mutation in familial and idiopathic PAH patients is on the advent. Gene-directed modulation of the BMPR2 receptor showed promise in experimental PH models. However, the use of CRISPR modulation as a pharmaceutical strategy, while a powerful tool, is not yet available for PAH patients.

In 2007: Reynolds et al. used an adenoviral vector for the targeted delivery of the BMPR2 gene to prevent BMPR2 inhibition in a rat model of hypoxia-induced PH. This treatment strategy significantly reduced the RVSP, RVH, and distal pulmonary vasculature muscularization [50]. However, the concern about a neutralizing immune response mounted after adenoviral transduction for horizontal gene transfer posed a concern for the efficacy of the method [51]. Building on these promising results, Harper et al. [52] improved established experimental PH in Monocrotaline (MCT) induced PH in rats using genetic modifications. Endothelial-like progenitor cells (ELPC) from the femural bone-marrow of rats were transduced with a BMPR2 adenoviral vector (AdCMVBMPR2myc) and were injected into the tail-vein of experimental PH rats. While the injected cells were short lived in the lungs (<24 h), the injected animals showed an immediate increase in BMPR2 in their lungs, which was thought to be exosome mediated, as well as an improvement in muscularized vessels over time [52]. Another avenue to overcome the obstacle of a neutralizing immune response would be the use of Adeno-associated virus (AAV) for gene delivery, which elicits only a neglectable immune response [53,54]. The use of the adenovirus for BMPR2 AAV1.SERCA2a reduced RV hypertrophy, RVSP, mPAP and vascular remodeling, thereby overall reducing experimental PH [55]. Currently, this strategy is investigated in translational studies for heart failure and PH [56]. The effect of the correction of a BMPR2 mutation by CRISPR was investigated by Gu et al. [39], where induced pluripotent stem cell-derived endothelial cells (iPSC-ECs) from different individuals amongst three single families were examined for their characteristics. Endothelial cells derived from FPAH patients were defective in cell survival, adhesion, migration, tube formation and BMPR2 signaling, whilst unaffected mutation carriers as well as CRISPR corrected iPSC-ECs were not.

As the presence of a BMPR2 mutation can reduce not only the functionality, but also the expression of the BMPR2 receptor, the induction of readthrough of nonsense mutations by ataluren has been employed to increase BMPR2 signaling in several lung and blood cell types [57]. Similarly, gentamycin was used to treat premature stop codons and readthrough in PAH [58,59].

### 5.2. Modulating the Availability of the BMPR2 Receptor at the Cell Surface

The increased availability of the BMPR2 receptor at the cell surface is a potent strategy to increase downstream BMPR2 signaling. We have shown that BMPR2 signaling can be modulated by upstream modifiers that we have targeted by repurposed pharmaceuticals [27]. In an siRNA high-throughput screen of over 20,000 genes of potential BMPR2 modulators, the tumor suppressor gene Fragile Histidine Triad (FHIT) was identified and thereafter pharmaceutically targeted by the repurposed drug Enzastaurin, which reversed established experimental PH in Sugen Hypoxia rats. Despite the established pharmacological role of Enzastaurin in PKC-inhibition, the authors showed that the action of Enzastaurin on BMPR2 signaling may likely be an unspecific effect of the drug, as other PKC inhibitors were unable to achieve the same effect on BMPR2 gene expression and PAEC function.

Similarly, treatment with the elastase-specific inhibitor elafin stabilized the BMPR2 receptor at the membrane in animal PH models by enhancing its interaction with Caveolin-1 and thus reversed established PH in Sugen-hypoxia rats and patient PAECs [60].

Likewise, the prevention of receptor degradation at the cell surface by chloroquine and hydro-chloroquine by inhibition of autophagy and lysosomal degradation prevented the development of experimental PH [61,62]. BMPR2 receptor degradation and receptor shedding is also targeted by the TNF-α antagonist Etanercept [37], which prevented and reversed experimental PH in rats [63] and endotoxic pigs [64].

Increasing BMPR2 shuttling to the membrane using the chaperone 4-phenylbutyric acid (4PBA) [65] led to a mild improvement in BMPR2 downstream signaling in patient fibroblasts that contained a specific inactivating mutation C118W, which served as a proof of concept for the applicability of this method in patients and an important step towards precision medicine in PAH [66].

### 5.3. Increasing Downstream Gene Transcription by Targeting BMPR2 Signaling or Interacting Pathways

Classical activation of BMPR2 signaling is achieved through ligand binding to BMPR2 receptor complexes. BMPR2/Alk1 heterocomplexes are mainly targeted by BMP9 [26], whereas other BMP ligands, such as BMP2 and BMP4, can activate multiple BMPR2 heterocomplexes (i.e., BMPR2/BMPR1A-B, BMPR2/Alk3), resulting in a higher probability for off-target effects in gene expression of bone formation signaling [67]. In PAECs, the administration of BMP9 prevents EC apoptosis consistent with the desired therapeutic outcome of preventing early vessel loss. Injection of the BMP9 ligand in mice and rats reversed established experimental PH (MCT-induced and SuHx) even in the presence of a heterozygous, inactivating BMPR2 mutation.

A different approach would be to interfere with pathways or molecules that inhibit the BMPR2 receptor or its pathway such as TGF-β signaling or the binding protein FKBP12. FKBP12 can be pharmaceutically targeted by FK506 and also FKVP, a non-immunosuppressive FK506 analog, which both activate BMPR2 signaling by FKBP12 antagonism [23,68]. The drug etanercept likewise increased BMPR2 signaling by inhibiting the BMP inhibitory pathway TGF-β, an effect that can also be observed with other TGF-β inhibitory substances, such as Paclitaxel [69].

Lastly, many pathways converge to induce BMPR2 signaling, opening the pharmaceutical potential of combined use of BMPR2-potentiating medication to achieve the synergistic, or additive activation of BMPR2 signaling. As a proof-of-concept, FK506 and Enzastaurin showed additive effects on BMPR2 signaling activation in vitro [27]. Moreover, the loss of BMPR2 leads to changes in several of its downstream signaling pathways, such as p38/MAPK/ERK [70], PI3K/Akt [71] and Wnt [72] signaling, which have thus also been investigated as therapeutic targets at a molecular level. Exploring the potential additive effects of targeting the BMPR2 receptor, as well as its downstream signaling, may be of therapeutical value.

## 6. Conclusions

PAH is a progressive and ultimately fatal disease, while current treatments are insufficient to substantially prolong patient survival. Targeting BMPR2 signaling and interacting signaling pathways has emerged as a promising approach to identify disease modifying therapies that address fundamental, genetically based molecular pathways important in PAH pathogenesis. Additive and synergistic effects of a combination treatment with several BMPR2 enhancing drugs have been shown to increase the therapeutic effect. However, the off-target effects of existing BMPR2-targeting pharmaceuticals hinder the precise assessment of the full potential of BMPR2 targeting in PAH therapy.

In summary, the use of BMPR2 targeted treatments in addition to conventional vasodilatory drugs in PAH is a promising avenue to explore in the search for novel PAH treatments, but the development of novel compounds to target BMPR2 signaling with increased specificity is of utmost importance.
